# Ambient temperature effect on pulse rate variability as an alternative to heart rate variability in young adult

**DOI:** 10.1007/s10877-015-9798-0

**Published:** 2015-10-28

**Authors:** Hangsik Shin

**Affiliations:** Department of Biomedical Engineering, Chonnam National University, Yeosu, South Korea

**Keywords:** Autonomous nervous system, Heart rate variability, Pulse rate variability, Temperature effect

## Abstract

Pulse rate variability (PRV) is a promising physiological and analytic technique used as a substitute for heart rate variability (HRV). PRV is measured by pulse wave from various devices including mobile and wearable devices but HRV is only measured by an electrocardiogram (ECG). The purpose of this study was to evaluate PRV and HRV at various ambient temperatures and elaborate on the interchangeability of PRV and HRV. Twenty-eight healthy young subjects were enrolled in the experiment. We prepared temperature-controlled rooms and recorded the ECG and photoplethysmography (PPG) under temperature-controlled, constant humidity conditions. The rooms were kept at 17, 25, and 38 °C as low, moderate, and high ambient temperature environments, respectively. HRV and PRV were derived from the synchronized ECG and PPG measures and they were studied in time and frequency domain analysis for PRV/HRV ratio and pulse transit time (PTT). Similarity and differences between HRV and PRV were determined by a statistical analysis. PRV/HRV ratio analysis revealed that there was a significant difference between HRV and PRV for a given ambient temperature; this was with short-term variability measures such as SDNN SDSD or RMSSD, and HF-based variables including HF, LF/HF and normalized HF. In our analysis the absolute value of PTT was not significantly influenced by temperature. Standard deviation of PTT, however, showed significant difference not only between low and moderate temperatures but also between low and high temperatures. Our results suggest that ambient temperature induces a significant difference in PRV compared to HRV and that the difference becomes greater at a higher ambient temperature.

## Introduction

The terms “pulse rate” and “heart rate” are frequently used interchangeably in the latest personalized wearable, health monitor devices. Most of these devices are designed to be worn on the wrist and acquire the volume pulse by an optical or impedance-based method. These pulse wave measures are called photo-plethysmography (PPG) [1], impedance-plethysmography (IPG) [2] or magneto-plethysmography (MPG) [3] according to the measuring principle. A pulse wave signal contains very rich information. It could become a standard pulse oximeter (measuring blood oxygen saturation) and its analysis methods could include additional features such as a reliable determination of respiratory activity [4]. A pulse wave measure is often used to estimate the heart rate variability (HRV). For wearable devices, acquired volume pulse wave has been used to derive pulse rate in terms of a heart rate. However, applications of pulse wave should be expanded as part of improving wearable healthcare technology.

When measuring heart rate variability (HRV) with pulse wave analysis, it is assumed that pulse rate is a substitute for heart rate. The term HRV refers to the variation of interbeat interval (IBI) [5, 6]. Even with controversies in using HRV clinically, HRV is proposed as a reliable and multifunctional parameter for cardiovascular and autonomic activities as well as a general measure of psychic and somatic fitness. The main focus of pulse wave research is in addressing whether the ECG-based method of determining HRV can be replaced by a technique that measures pulse wave. Pulse wave is generated by cyclic cardiac activity of systole and diastole and as such, it reflects the cardiac cycle, but pulse wave can be affected by vascular characteristics. A pulse wave based analysis of cardiac interval is called pulse rate variability (PRV), and PRV is evaluated by the same methods as for HRV in general.

A number of studies have evaluated whether PRV could be a surrogate for HRV [7–11]. In these studies, an assessment such as SDNN has been used; SDNN denotes the standard deviation of normal to normal R–R intervals (NN), where R is the peak of a QRS complex in an ECG recording of a heartbeat. Additional parameters like root mean square of successive difference between adjacent NN intervals (RMSSD), proportion of NN50 in total NN intervals (pNN50), low frequency (LF) power, high frequency (HF) power or LF to HF ratio (LF/HF) have also been used. Values derived for HRV and PRV were then compared statistically to evaluate interchangeability. From these studies, the agreement between HRV and PRV was mostly acceptable for a test subject at sitting, resting position. However, physical position of a subject (like standing or being in an upright tilt versus resting in a supine position) can result in PRV divergence from HRV. This is because PRV is a reflection of the mechanical coupling between respiration and thoracic vascular systems and is stronger at a standing position than at a supine one [12]. From this observation PRV can only be recommended as a surrogate for HRV at a resting position.

Another factor that may cause a divergence of PRV from HRV is a subject’s mental state as it can lead to changes in vasomotor activity. Research has shown that mental stress can lead to increases in atrial stiffness which affects pulse wave velocity (PWV) [13]. A change in PWV could then, in turn, induce variations not only in pulse transit time (PTT) but also PRV. The purpose of this study was to evaluate the effect of ambient temperature on HRV and PRV and gain a better understanding of behavior of PRV with respect to HRV with the external parameter of temperature. We used photo-plethysmography (PPG) for detection of PRV.

## Methods

**Fig. 1:**
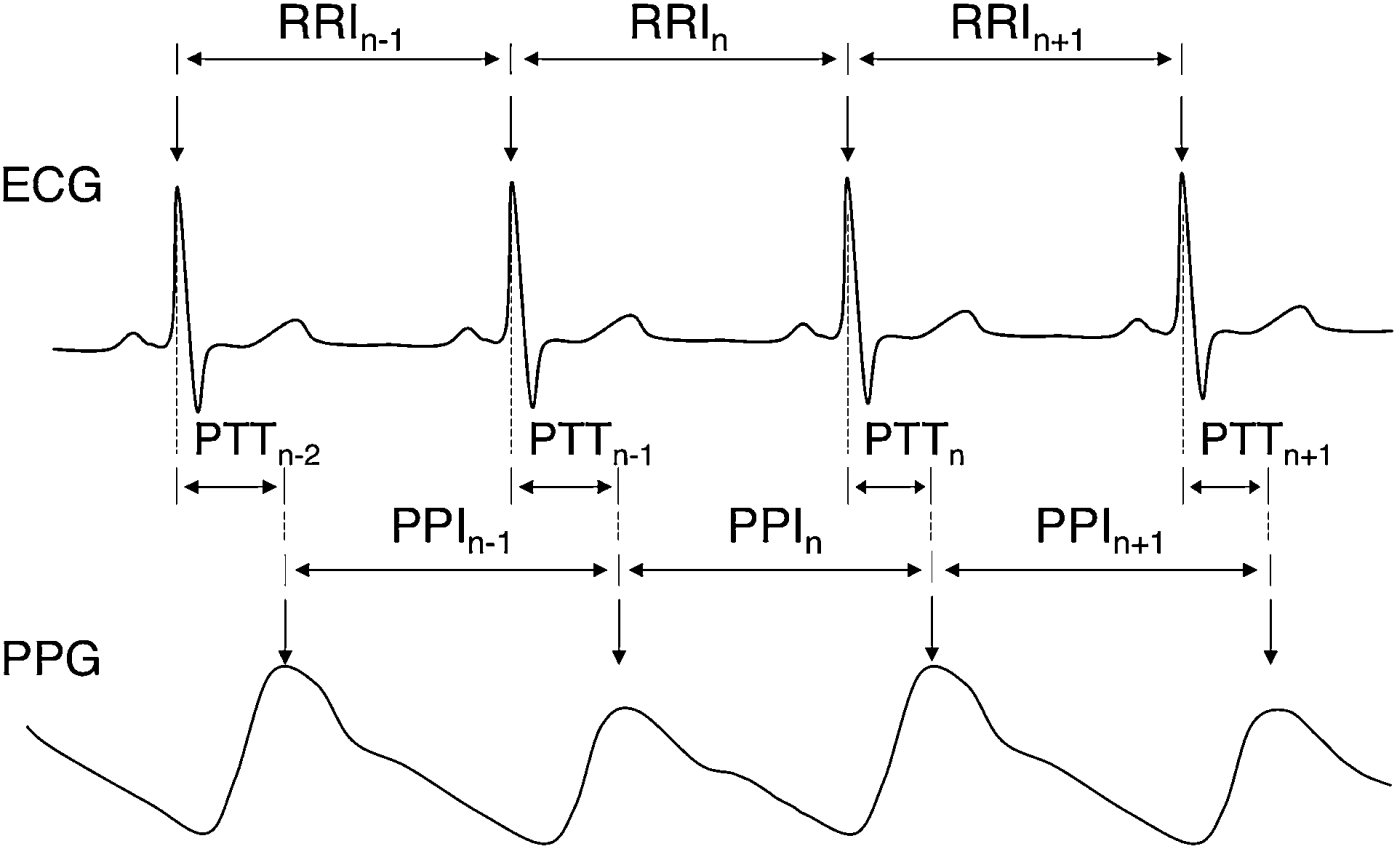
ECG and PPG waveform and beat-related features

HRV is derived from a series of IBIs between two QRS complexes in a heart ECG wave. In the same manner, peak-to-peak intervals (PPIs) of upper peaks or lower peaks are used to derive the PRV with photo-plethysmography (PPG) [14]. In our research, Pan and Tompkins algorithm [15] and adaptive threshold peak detection algorithm [16] were used to detect the QRS complex of ECG waveform and upper and lower peak of a PPG waveform, respectively. Representative features used in HRV or PRV analysis are shown in Fig. 1. In Fig. 1, PTT is the time it takes for a pulse wave to arrive at the measuring site after an electrical activation of ventricle.

In time-domain analysis, we assessed multiple variables of HRV analysis [5, 6]; average NN interval (AVNN), standard deviation of NN interval (SDNN), standard deviation of successive difference between adjacent NN intervals (SDSD), root mean square of successive difference between adjacent NN intervals (RMSSD), the number of pairs of successive NNs that differ by more than 50 ms (NN50), and proportion of NN50 in total NN intervals (pNN50). Variables for long-term HRV monitoring such as the mean of the 5-min standard deviation of the NN interval (SDNN index) and standard deviation of average NN intervals (SDANN) were excluded in this study as we focused on short-term physiological activities.

For frequency domain analysis, we preprocessed the signals to obtained IBIs. After detecting all peaks, we interpolated IBI series with 1 kHz sampling rate first. Then we resampled with a 4 Hz sampling frequency. In transforming the signal to the frequency domain, band-limited characteristic of the time series caused signal discontinuity. This discontinuity could produce an additional side lobe around the signal frequency and cause the loss of frequency power. We used a Hanning window with the same length to the IBI series to obtain a more accurate frequency component for the HRV and PRV frequency analysis. Then, we use the fast Fourier Transform (FFT) to calculate frequency domain component. For assessment of HRV and PRV on frequency domain, we measured and assembled the values of very low frequency (VLF, 0.0033 ~ 0.04 Hz), low frequency (LF, 0.04 ~ 0.15 Hz), high frequency (HF, 0.15 ~ 0.5 Hz), LF/HF ratio (LF/HF), normalized LF (nLF), and normalized HF (nHF).

To compare the temperature effects on HRV and PRV values, we derived the comparator parameter PRV/HRV ratio for each variable. This allowed us to investigate the effects of temperature stress on PRV/HRV ratios. The PRV/HRV ratio is defined as *Var*_*R*_ = *Var*_*PRV*_/*Var*_*HRV*_ where *Var* represents the variability of time and frequency domains under study. We also analyzed PTT and standard deviation of PTT (SDPTT) as a vasomotor index according to the temperature change. PTT was derived from the time difference between the QRS complex and the upper peak of PPG (see Fig. 1). For this, we synchronized the QRS and upper peak of PPG as manual.

### Experimental design

**Fig. 2:**
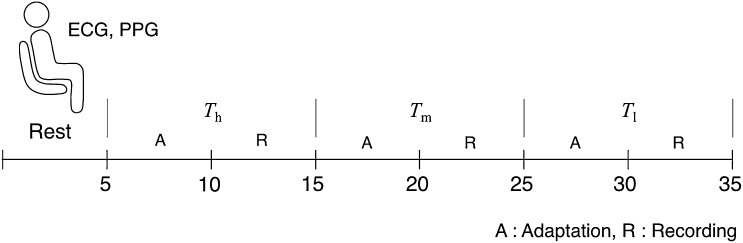
Experimental protocol

Three rooms were set to different ambient temperatures. The room temperature was controlled to a relatively low (*T*_l_ = 17 °C), moderate (*T*_m_ = 25 °C), or a high temperature (*T*_h_ = 38 °C) by air conditioning systems and heating devices. These settings were after considering the thermal comfort standard recommended by the ISO 7730 standard [17]. Humidity was controlled around a relative humidity (RH) of 30 % (27 ± 5 %) as this level of humidity does not affect a young adult physiology after a short exposure [18]. Experiments were conducted for 10 min each per condition. To provide an adaptation time to each temperature, data from the first 5-min exposure were excluded in the analysis. Before each experiment, height, weight, and blood pressure measures were obtained for each participant. The body mass index (BMI) was calculated for each participant (using the formula BMI = mass (in ‘kg’)/[height (in ‘m’)]^2^). For a synchronized analysis of PRV and HRV, ECG and PPG were recorded simultaneously for each subject. Ambient room temperature was kept constant throughout the recordings. All the data was recorded in a sitting position. Talking and movement were restricted and each participant wore typical street cloths. The experimental sequence is described in Fig. 2. For our measurements, we calculated the parameters elaborated above for each temperature setting.

### Statistical analysis

Paired *t* test was used for evaluating the differences between the HRV and PRV parameters for each temperature condition. One-way ANOVA test was carried out for each variable to evaluate the variables between various temperatures. As a post hoc test, when the variances can be assumed to be equal, we used Bonferroni’s test. Otherwise, we used Tamhane’s T2 test for the post hoc test. Each variable was calculated using Mathworks MATLAB R2014a (Mathworks, Natica MA, USA). SPSS version 21.0 was used for statistical analysis (IBM, Armonk NY, USA).

## Results

### Participants

Twenty-eight healthy young subjects (11 women, 17 men) participated in this study. Each subject voluntarily participated in this study and each provided a consent. Their age, height, weight, and body mass index (BMI) were 20.8 ± 1.0 years, 169.5 ± 7.38 cm, 63.5 ± 13.9 kg, and 22.0 ± 3.7 kg/m^2^, respectively. The participants were healthy with no cardiovascular or respiratory conditions to consider. To avoid unwanted effects on the autonomic nervous system activity, the participants agreed to abstain from drinking, smoking, intense exercise, and caffeine intake the day before the experiment.

### HRV and PRV measures at various ambient temperatures

**Fig. 3:**
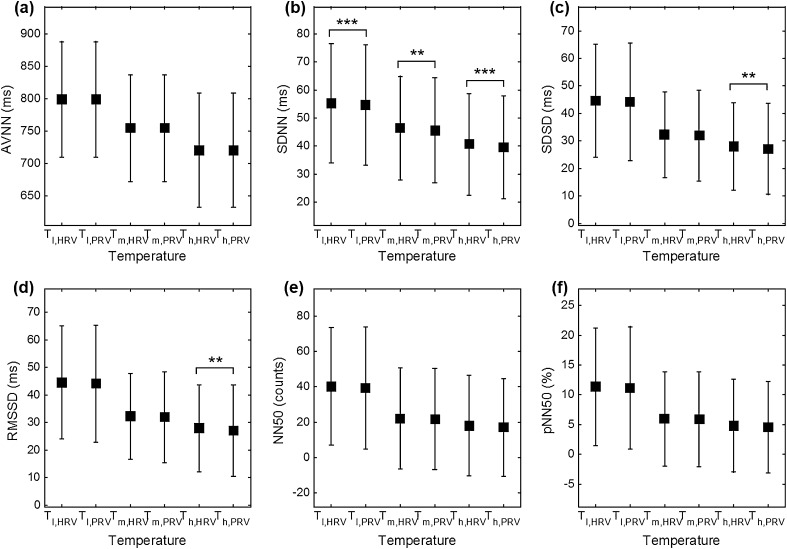
Results comparison between HRV and PRV on time-domain. **a** AVNN, **b** SDNN, **c** SDSD, **d** RMDDS, **e** NN50 and **f** pNN50 (***P < 0.001, **P < 0.01 and *P < 0.05)

**Fig. 4:**
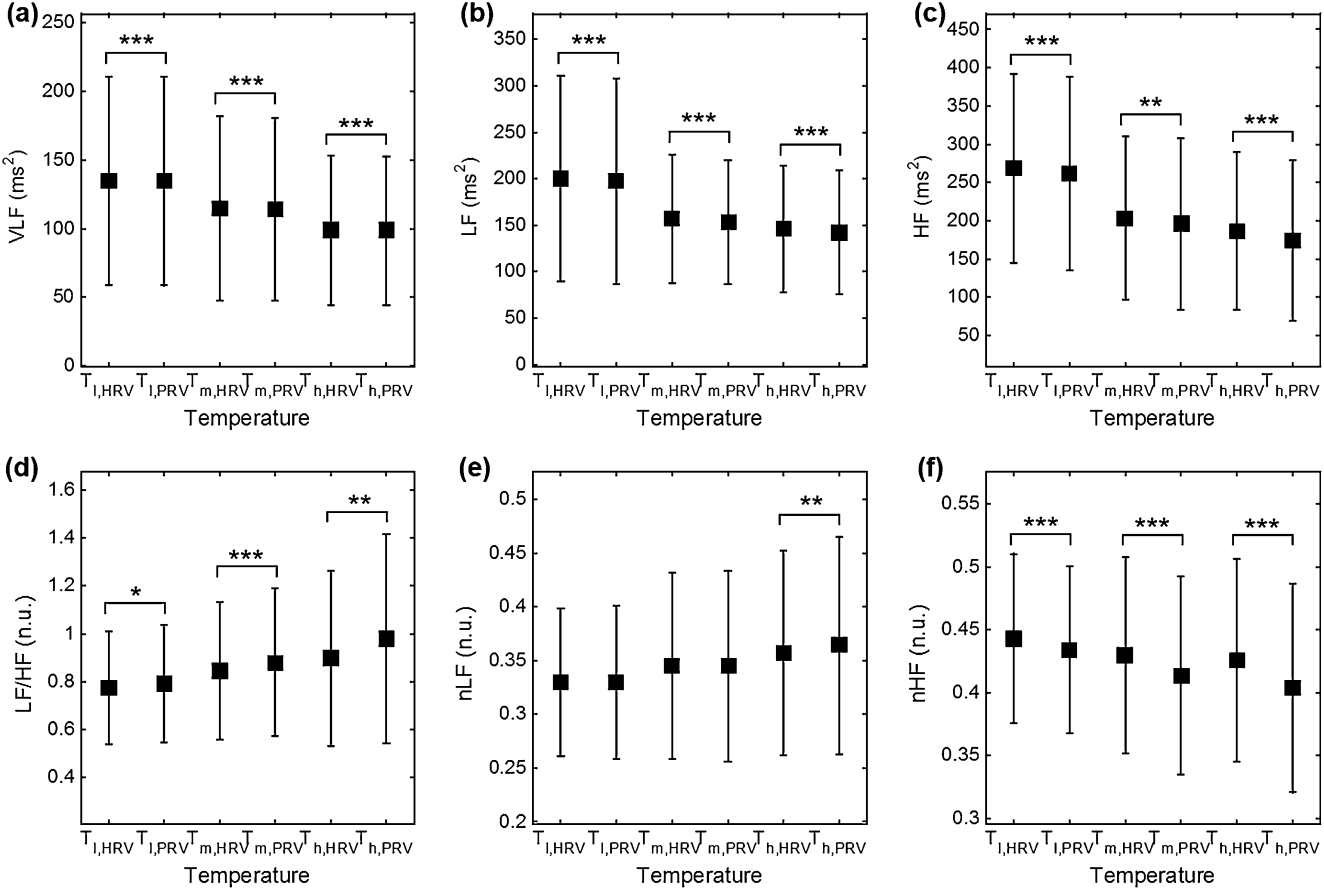
Results comparison between HRV and PRV on frequency-domain **a** VLF, **b** LF, **c** HF, **d** LF/HF, **e** nLF and **f** nHF (***P < 0.001, **P < 0.01 and *P < 0.05)

Values of HRV and PRV for specific temperature conditions are summarized in Table 1. In time-domain analysis, the AVNN, NN50, and pNN50 values were not significantly different for each temperature condition. However, SDNN, SDSD, and RMSSD showed significant differences. SDNN, SDSD, and RMSSD commonly showed a significant difference at *T*_h_ = 38 °C, and SDNN showed significance in every temperature condition. In frequency analysis, significant differences were observed in every case except for the nLF. Graphical representations of HRV and PRV analysis are shown in Fig. 3 for the time-domain (AVNN, SDNN, SDSD, RMDDS, NN50 and pNN50) and in Fig. 4 for the frequency-domain analysis (VLF, LF, HF, LF/HF, nLF and nHF).

**Table 1:** Results of each variable and significance between HRV and PRV analysis

Variable	Method	Temperature condition (mean ± SD)		
		*T*_l_	*T*_m_	*T*_h_
AVNN (ms)	HRV	799.1 ± 89.0	754.6 ± 82.1	720.8 ± 87.8
	PRV	799.1 ± 89.0	754.6 ± 82.2	720.8 ± 87.8
*P* value		0.093	0.103	0.249
SDNN (ms)	HRV	54.7 ± 21.5	45.7 ± 18.7	39.6 ± 18.4
	PRV	55.3 ± 21.3	46.4 ± 18.5	40.6 ± 18.2
*P* value		***	**	***
SDSD (ms)	HRV	44.2 ± 21.3	31.9 ± 16.5	27.1 ± 16.5
	PRV	44.6 ± 20.5	32.2 ± 15.6	28.0 ± 15.8
*P* value		0.088	0.434	**
RMSSD (ms)	HRV	44.1 ± 21.3	31.9 ± 16.4	27.1 ± 16.5
	PRV	44.5 ± 20.5	32.2 ± 15.5	27.9 ± 15.8
*P* value		0.088	0.434	**
NN50 (sample)	HRV	39.3 ± 34.5	21.8 ± 28.6	17.0 ± 27.5
	PRV	40.2 ± 33.3	22.1 ± 28.7	18.1 ± 28.3
*P* value		0.182	0.555	0.084
pNN50 (%)	HRV	11.2 ± 10.3	5.9 ± 8.0	4.6 ± 7.7
	PRV	11.3 ± 9.9	6.0 ± 7.9	4.8 ± 7.8
*P* value		0.318	0.608	0.105
VLF (ms^2^)	HRV	134.6 ± 76.0	114.2 ± 66.8	98.5 ± 54.3
	PRV	135.0 ± 76.1	114.6 ± 67.1	98.8 ± 54.5
*P* value		***	***	***
LF (ms^2^)	HRV	197.2 ± 110.6	153.3 ± 66.8	142.1 ± 66.9
	PRV	200.2 ± 110.8	156.6 ± 69.0	145.5 ± 68.2
*P* value		***	***	***
HF (ms^2^)	HRV	261.4 ± 126.2	196.1 ± 111.9	174.0 ± 105.1
	PRV	268.3 ± 123.2	203.8 ± 106.6	186.5 ± 103.0
*P* value		***	**	***
LF/HF (n.u.)	HRV	0.790 ± 0.246	0.880 ± 0.308	0.979 ± 0.437
	PRV	0.774 ± 0.235	0.844 ± 0.287	0.897 ± 0.367
*P* value		*	***	**
nLF (n.u.)	HRV	0.330 ± 0.071	0.345 ± 0.089	0.364 ± 0.101
	PRV	0.330 ± 0.069	0.345 ± 0.087	0.357 ± 0.095
*P* value		0.928	0.852	**
nHF (n.u.)	HRV	0.434 ± 0.066	0.414 ± 0.079	0.404 ± 0.083
	PRV	0.443 ± 0.067	0.430 ± 0.078	0.426 ± 0.081
*P* value		***	***	***

*** *P* < 0.001, ** *P* < 0.01 and * *P* < 0.05

### Comparison of HRV and PRV analysis in temperature changes

**Fig. 5:**
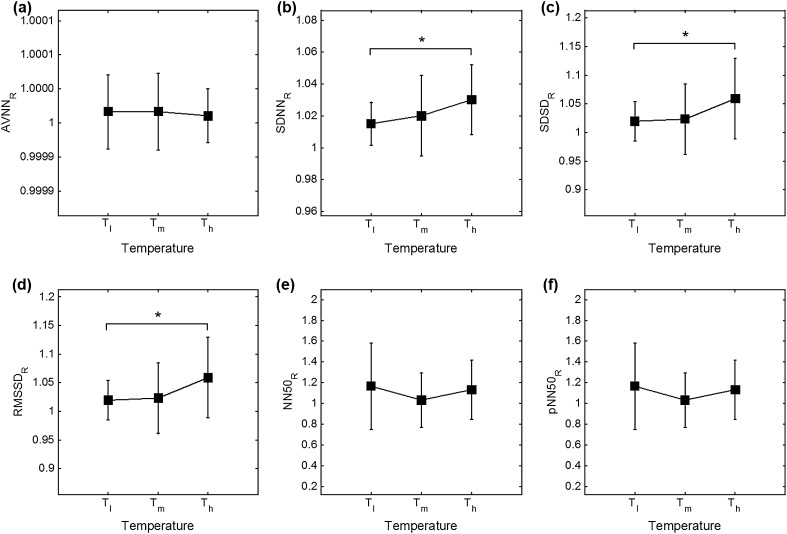
Results of the significance analysis of HRV–PRV ratio between temperature conditions on time-domain, **a** AVNN_R_, **b** SDNN_R_, **c** SDSD_R_, **d** RMSSD_R_, **e** NN50_R_ and **f** pNN50_R_. (*P < 0.05)

**Fig. 6:**
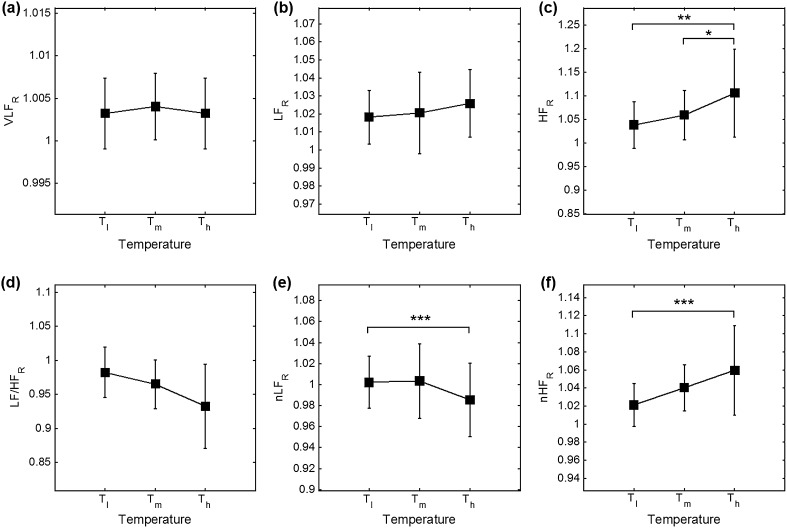
Results of the significance analysis of HRV–PRV ration between temperature conditions on frequency-domain, **a** VLF_R_, **b** LF_R_, **c** HF_R_, **d** LF/HF_R_, **e** nLF_R_ and **f** nHF_R_. (***P < 0.001, **P < 0.01 and *P < 0.05)

**Fig. 7:**
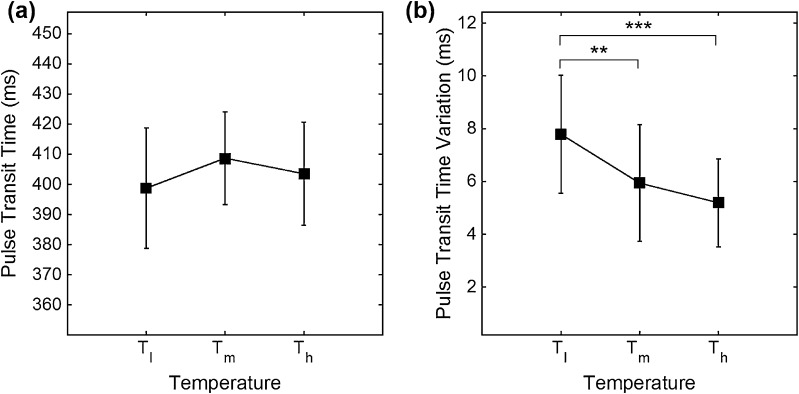
Results of the significance analysis of PTT and SDPTT analysis between temperature conditions, **a** PTT and **b** SDPTT. (***P < 0.001 and **P < 0.01)

In evaluating the temperature effect on differences between HRV and PRV, we derived the PRV/HRV ratio of each variable (AVNN_R_, SDNN_R_, SDSD_R_, RMSSD_R_, NN50_R_, pNN50_R_, VLF_R_, LF_R_, HF_R_, LF/HF_R_, nLF_R_ and nHF_R_). These variables were subjected to one-way ANOVA with post hoc test to validate the significance according to the analytic methods. Values and differences of variables according to the temperature change are summarized in Table 2. In our analysis, significance was found in SDNN_R_, SDSD_R_, RMSSD_R_, HF_R_, LF/HF_R_, nHF_R_ ratios and SDPTT. For the temperatures analyzed, the highest significance was found for *T*_l_–*T*_h_. The significance in *T*_*m*_–*T*_*h*_ and in *T*_*l*_–*T*_*m*_ was only for HF_R_ and SDPTT, respectively. PTT, as the vasomotor index, was not significantly influenced by temperature. However, SDPTT showed significant change for *T*_l_–*T*_m_ and *T*_l_–*T*_h_ cases. Results of the significance analysis of PRV/HRV ratio for temperature conditions on time-domain and frequency domain are shown in Figs. 5 and 6, respectively. Figure 7 represent the PTT and SDPTT for the temperature conditions graphically.

**Table 2:** Results of each variable and significance between HRV and PRV analysis

Variable (unit)	Temperatures	Mean ± SD	Temperature difference	Mean difference
AVNN_R_^a^ (n.u.)	*T*_l_	1.000 ± 0.000	*T*_l_–*T*_m_	0.000
	*T*_m_	1.000 ± 0.000	*T*_m_–*T*_h_	0.000
	*T*_h_	1.000 ± 0.000	*T*_l_–*T*_h_	0.000
SDNN_R_^b^ (n.u.)	*T*_l_	1.015 ± 0.013	*T*_l_–*T*_m_	−0.005
	*T*_m_	1.020 ± 0.025	*T*_m_–*T*_h_	−0.010
	*T*_h_	1.030 ± 0.022	*T*_l_–*T*_h_	−0.015*
SDSD_R_^b^ (n.u.)	*T*_l_	1.020 ± 0.035	*T*_l_–*T*_m_	−0.004
	*T*_m_	1.023 ± 0.062	*T*_m_–*T*_h_	−0.036
	*T*_h_	1.059 ± 0.070	*T*_l_–*T*_h_	−0.040*
RMSSD_R_^b^ (n.u.)	*T*_l_	1.020 ± 0.035	*T*_l_–*T*_m_	−0.004
	*T*_m_	1.023 ± 0.062	*T*_m_–*T*_h_	−0.036
	*T*_h_	1.059 ± 0.070	*T*_l_–*T*_h_	−0.040*
NN50_R_^a^ (n.u.)	*T*_l_	1.166 ± 0.416	*T*_l_–*T*_m_	0.133
	*T*_m_	1.033 ± 0.261	*T*_m_–*T*_h_	−0.100
	*T*_h_	1.133 ± 0.286	*T*_l_–*T*_h_	0.033
pNN50_R_^a^ (n.u.)	*T*_l_	1.166 ± 0.416	*T*_l_–*T*_m_	0.133
	*T*_m_	1.033 ± 0.261	*T*_m_–*T*_h_	−0.100
	*T*_h_	1.133 ± 0.286	*T*_l_–*T*_h_	0.033
VLF_R_^a^ (n.u.)	*T*_l_	1.003 ± 0.004	*T*_l_–*T*_m_	−0.001
	*T*_m_	1.004 ± 0.004	*T*_m_–*T*_h_	0.001
	*T*_h_	1.003 ± 0.004	*T*_l_–*T*_h_	−0.000
LF_R_^a^ (n.u.)	*T*_l_	1.018 ± 0.015	*T*_l_–*T*_m_	−0.002
	*T*_m_	1.021 ± 0.023	*T*_m_–*T*_h_	−0.005
	*T*_h_	1.026 ± 0.019	*T*_l_–*T*_h_	−0.008
HF_R_^b^ (n.u.)	*T*_l_	1.038 ± 0.049	*T*_l_–*T*_m_	−0.021
	*T*_m_	1.059 ± 0.052	*T*_m_–*T*_h_	−0.047*
	*T*_h_	1.106 ± 0.093	*T*_l_–*T*_h_	−0.068**
LF/HF_R_^b^ (n.u.)	*T*_l_	0.982 ± 0.037	*T*_l_–*T*_m_	0.017
	*T*_m_	0.965 ± 0.036	*T*_m_–*T*_h_	0.032
	*T*_h_	0.933 ± 0.062	*T*_l_–*T*_h_	0.050***
nLF_R_^a^ (n.u.)	*T*_l_	1.002 ± 0.025	*T*_l_–*T*_m_	−0.001
	*T*_m_	1.003 ± 0.035	*T*_m_–*T*_h_	0.018
	*T*_h_	0.986 ± 0.035	*T*_l_–*T*_h_	0.017
nHF_R_^b^ (n.u.)	*T*_l_	1.021 ± 0.024	*T*_l_–*T*_m_	−0.019
	*T*_m_	1.040 ± 0.025	*T*_m_–*T*_h_	−0.019
	*T*_h_	1.060 ± 0.049	*T*_l_–*T*_h_	−0.038***
PTT^a^ (ms)	*T*_l_	399.8 ± 20.1	*T*_l_–*T*_m_	−9.951
	*T*_m_	408.7 ± 15.4	*T*_m_–*T*_h_	4.761
	*T*_h_	402.6 ± 17.1	*T*_l_–*T*_h_	5.189
SDPTT^b^ (ms)	*T*_l_	7.8 ± 2.2	*T*_l_–*T*_m_	1.851**
	*T*_m_	5.9 ± 2.2	*T*_m_–*T*_h_	0.745
	*T*_h_	5.2 ± 1.7	*T*_l_–*T*_h_	2.596***

^a^Bonferonni’s post hoc test

^b^Tamhane’s T2 post hoc test

*** *P* < 0.001, ** *P* < 0.01 and * *P* < 0.05

## Discussion

External temperature can affect physiological status and understanding the role of ambient temperature on heart function based on a PRV read could be useful in better utilizing the pulse-wave for healthcare applications. As most PRV-based applications will be used in ambulatory situations, it is important to know how ambient temperature could affect PRV. Discrepancies between PRV and HRV come from physiological processes rather than from noise or artifacts even though an ambulatory monitoring of pulse rate may have inaccuracies [19]. From Gil et al.’s [20] research, the bias between PRV and HRV could not be explained by random fluctuations in peak detection.

Schafer and Vagedes [14] postulated that the pulse transit time (PTT) might be mainly responsible for the differences in PRV and HRV peak detection. Variability in PTT is induced, for example, by respiratory activity. Respiration-induced change is one of representative factors that can change IBIs. Respiration could change the intra-thoracic pressure, thus causing blood flow variations in the venous circulation [21] as well as in the arterial system [22]. For example, inspiration can decrease the intra-thoracic pressure, thus reducing the left ventricular stroke volume as well as the pressure and cross-section of the thoracic aorta [23]. Such passively induced changes of cardiovascular loading may be accompanied by locally modulated spontaneous oscillations of small arteries. Moreover, other sympathetically controlled vasomotor activities occur independently at low frequencies (below 0.15 Hz) [24]. As such, these phenomena may change the transit time of pulse from the heart to the measuring site, causing differences between HRV and PRV (in the LF domain). Respiration-induced variations of LF power are less significant at a resting condition. However, it could make considerable bias in LF/HF or normalized LF with underestimations of HRV with PRV reads. This activity could also explain why PRV could overestimate HRV mostly in HF domain or in short-term variability such as HF, RMSSD, and pNN50. Our results represent HRV and PRV characteristics for a specified temperature. It was shown that PRV is overestimated in most of frequency-domain analysis results except for LF/HF and in short-term variability related variables of time-domain analysis such as SDNN, SDSD and RMSSD. For LF/HF, PRV overestimated HRV. These under- or over-estimations of PRV have been shown in previous studies [25].

Our results indicate that the difference between HRV and PRV is significantly increased at higher ambient temperatures. PRV/HRV ratio analysis clearly affirms this temperature effect. Our results also show that the deviation of IBI is increased at a higher ambient temperature environment. This may be caused by more vasodilation at higher temperatures, changing the blood flow in small arteries and causing variations in PTT by baroreflex activity. For example, SDPTT significantly decreased at the higher temperature recorded (Fig. 7b), which could be a reflection of changes in vasomotor activity. In contrast, at low ambient temperature, significant differences were rarely found between HRV and PRV in the time domain analysis.

From the above results, we summarize the HRV and PRV changes with temperature as follows: Differences between PRV and HRV are greater at a high temperature. For example a decrease in SDPTT at high ambient temperature could reflect decreased vasomotor activity. Moreover, differences between HRV and PRV brought on by temperature stress are more dramatic in HF component of HRV which concerns cardiac parasympathetic activity or respiratory sinus arrhythmia (RSA) [26].

According to previous studies on HRV–PRV relationship, PRV measure is a useful alternative to an HRV measure. However, there are some limitations to this assumption. As shown in the previous studies, physical activity and some mental stressors can impair the agreement between PRV and HRV, and often to an inacceptable extent. Moreover, the effects of sensor position or performance of detection algorithm were shown to cause variability. Also environmental effects such as ambient temperature or light on PRV measures had not been previously studied and as such, our study chose to measure the effect of ambient temperature on PRV reads. Our analysis was done in three different ambient temperature environments.

Our research has some limitations. First, every subject who participated in the study was a young healthy adult. Research based on various age groups is required for a more generalized result. Second, the effect of seasons or effect of length of adaptation time could have been explored in our study. Third, in our study, the respiration rates of the participants were not recorded or studied and every subject could breathe spontaneously. As mentioned above, respiration rate besides physical or mental factors could affect PRV. In our opinion, for a more complete HRV and PRV analysis, similar research may performed to investigate and quantify the influence of factors such as physical/physiological stress, use of drugs such as vasodilating drugs, and presence of neuropathic diseases such as diabetic neuropathy on PTT.

## Conclusion

Home, mobile or wearable healthcare solutions use have increased and will continue to increase as will the extent of personal health monitoring and care. Especially, most wearable devices include a heart monitor in form of a pulse detector. Because a pulse detector could measure pulse rate with simple hardware coupled with a processing algorithm, pulse wave based applications are being developed to provide a wealth of individual health information.

From this research, we found that the ambient temperature could induce a difference between HRV and PRV. The differences were found in the short-term variables that reflect the parasympathetic activity. Although this research has ambiguities such as the quantification of the temperature effect and levels of respiration, we believe that this research will improve our knowledge on how ambient temperature could affect PRV and will provide in designing better personal health monitors as part of mobile, personal care technologies.
